# Pichinde Virus Infection of Outbred Hartley Guinea Pigs as a Surrogate Animal Model for Human Lassa Fever: Histopathological and Immunohistochemical Analyses

**DOI:** 10.3390/pathogens9070579

**Published:** 2020-07-16

**Authors:** Wun-Ju Shieh, Shuiyun Lan, Sherif R. Zaki, Hinh Ly, Yuying Liang

**Affiliations:** 1 Infectious Disease Pathology Branch, Centers for Disease Control and Prevention, Atlanta, GA 30333, USA; sxz1@cdc.gov; 2 Department of Pathology and Laboratory Medicine, Emory University, Atlanta, GA 30322, USA; shuiyun.lan@emory.edu; 3 Department of Veterinary and Biomedical Sciences, College of Veterinary Medicine, University of Minnesota, St Paul, MN 55108, USA

**Keywords:** mammarenavirus, arenavirus, Pichinde virus, Lassa virus, animal model, pathology, virulence, histopathology, immunohistochemistry, pathogenesis, surrogate animal model

## Abstract

Lassa virus (LASV) is a mammarenavirus (arenavirus) that causes zoonotic infection in humans that can lead to fatal hemorrhagic Lassa fever (LF) disease. Currently, there are no FDA-approved vaccines or therapeutics against LASV. Development of treatments against LF and other related arenavirus-induced hemorrhagic fevers (AHFs) requires relevant animal models that can recapitulate clinical and pathological features of AHF diseases in humans. Laboratory mice are generally resistant to LASV infection, and non-human primates, while being a good animal model for LF, are limited by their high cost. Here, we describe a small, affordable, and convenient animal model that is based on outbred Hartley guinea pigs infected with Pichinde virus (PICV), a mammarenavirus that is non-pathogenic in humans, for use as a surrogate model of human LF. We conducted a detailed analysis of tissue histopathology and immunohistochemical analysis of different organs of outbred Hartley guinea pigs infected with different PICV strains that show differential disease phenotypes and pathologies. Comparing to infection with the avirulent PICV strain (P2 or rP2), animals infected with the virulent strain (P18 or rP18) show extensive pathological changes in different organs that sustain high levels of virus replication. The similarity of tissue pathology and viral antigen distribution between the virulent PICV–guinea pig model and lethal human LASV infection supports a role of this small animal model as a surrogate model of studying human LF in order to understand its pathogenesis and for evaluating potential preventative and therapeutic options against AHFs.

## 1. Introduction

Lassa virus (LASV), the causative agent of Lassa fever (LF), was first isolated from two missionary nurses who died from LF in Nigeria in 1969 [1,2]. LASV causes zoonotic infection in humans through ingestion of contaminated food and/or water, inhalation of tainted aerosols, or via person-to-person contact with infected body fluids [2]. New LASV infected cases each year are estimated to be up to 500,000 with 5000 deaths [2,3]. While the average case fatality rate of LF is ~1%, it can reach as high as 20% in hospitalized patients [2]. Currently, there are no FDA-approved vaccines or therapeutics against LASV. Development of treatments against LASV infection requires relevant animal models that can recapitulate disease and pathological features of LF and other AHFs in humans.

The description of different animal models of LF has recently been reviewed elsewhere [4]. Each of the known LF animal models has its advantages and disadvantages. For example, nonhuman primates (NHPs) infected with a virulent strain of LASV can develop a severe form of the disease that is similar to human LF, and thus are deemed the “gold standard” models for use in pathogenesis studies as well as for vaccine and antiviral testing. However, limitations of using NHPs include the extremely high cost and the requirement for high containment BSL4 facilities to conduct LASV studies. Laboratory mice, on the other hand, are relatively inexpensive, yet they are generally resistant to LASV infection and do not develop obvious diseases [5]. However, LASV infection of the 129Sv mice, which lack the receptor for interferon α and β (IFNAR^-/-^), can lead to a non-lethal acute infection with persistent viremia, but do not develop febrile or neurological symptoms [6,7,8,9]. Likewise, mice lacking interferon α, β, and γ receptors (IFN α/β/γR^-/-^) did not develop clinical signs of disease upon LASV infection [8]. In contrast, LASV infection of STAT1^-/-^ mice showed marked body weight loss and either died or met humane endpoint criteria [9,10]. Recent development of different chimeric mouse models of LASV infection, such as a chimeric IFNAR^-/-B6^ lethal mouse model, in which the IFNAR^-/-^ mice were irradiated and transplanted with bone marrow progenitor cells from wild type C57BL/6 mice, showed great promise, as these chimeric mice could uniformly succumb to LASV infection while maintaining a normal immune function [6,11]. Additionally, a humanized HHD mouse model, which consists of C57BL/6 mice expressing a human/mouse chimeric HLA-A2.1 instead of the normal MHC class I gene products, has recently been developed for LASV infection that also shows some clinical disease similar to human LF [12]. While some of the mouse models appear to show LF-like clinical features, they are either immuno-incompetent or have been manipulated immunologically, making it challenging to model the natural immune responses to LASV infection and/or for use in vaccine development.

Immunologically competent inbred Strain 13 guinea pigs can be considered as an alternative small rodent model for LF, as LASV-infected animals show many of the clinical features of LF in humans [13,14,15]. It is important to note, however, that while inbred guinea pigs may be a useful model for LF, they are not readily available and lack genetic diversity mimicking human populations. On the contrary, outbred Hartley guinea pigs are commercially available and have diverse genetic background. An outbred LASV-infected Hartley guinea pig model has been developed that shows uniform lethality and clinical symptoms, such as fever, followed by hypothermia just before death, as well as weight loss, lethargy, thrombocytopenia, neutropenia, and lymphopenia [16].

Several surrogate animal models of LF using a less pathogenic virus within the same Arenaviridae family as LASV have been developed. While the lymphocytic choriomeningitis virus (LCMV) in general does not induce a severe LF-like disease in rodents or NHPs, a particular strain LCMV-WE has been used as a surrogate for LASV in rhesus macaques [17] and in HHD mice [18] that can model certain aspects of LASV pathogenesis such as vascular leak but cannot reproduce many characteristics of LF disease. Due to its pathogenicity in NHPs, work with LCMV-WE requires a BSL3 facility, which limits its usage in general laboratories. Pichinde virus (PICV) isolated from rice rats in Colombia is not known to cause disease in humans (non-pathogenic) [19]. Sequential passage of PICV in guinea pigs produced a virulent strain, which causes LF-like disease in strain 13 guinea pigs that reproduces many characteristics of LF such as fever, bleeding disorder, and general immune suppression, and therefore has been used as a safe and relevant surrogate human LF model in BSL2 facilities [20,21,22]. A minimum of four sequential passages of PICV in guinea pigs was required to result in 100% uniform lethality [22,23]. Infected guinea pigs showed extreme leukopenia and a transient neutrophilia [22]. Serologic findings include impaired platelet function, decreased activity of coagulation factors, and thrombocytopenia; decreased white blood cell levels; and increased hematocrit [23]. Histopathologic analysis indicates lesions in the liver, spleen, pancreas, lungs, and gastrointestinal tract [20,21,22,23]. Scattered necrotic regions have also been found in lymphoid tissue and the bone marrow [23].

We report here a detailed histopathologic and immunohistochemical (IHC) analyses of a wide range of tissues collected from organs of outbred Hartley guinea pigs that have been infected with PICV strains with the propensity to cause varied disease pathologies. Our study shows that virulent PICV strain (P18 or rP18) can replicate to high levels in various extraneuronal tissues and lead to extensive pathological changes. The similarity of tissue pathology and viral antigen distribution between the virulent PICV-guinea pig model and lethal human LF infection supports this outbred guinea pig model as a relevant surrogate animal model for use to characterize LASV infection and to study LF pathogenesis in vivo.

## 2. Results

### 2.1. Overview of the Viral Strains Used and the Disease Symptoms Produced by Them

**Figure 1:**
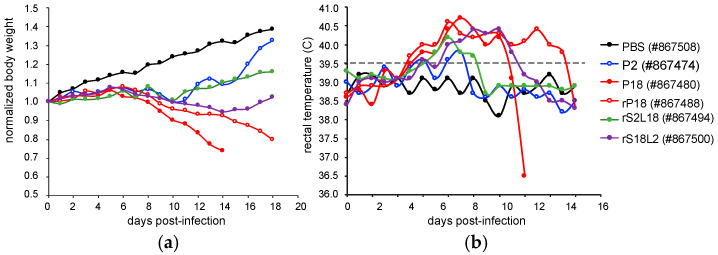
Body weight losses and increased rectal temperatures of outbred Hartley guinea pigs infected with the virulent P18 or rP18 strain of PICV as compared to those of animals infected with the avirulent P2 or rP2 strain. Outbred Hartley guinea pigs were either mock infected (with PBS) or infected with various PICV strains (P2, rP2, P18, or rP18). Each animal was monitored daily for body weight loss (normalized to day 0) (**A**) and rectal temperature change (**B**). Fever is defined as rectal temperature of >39.5 °C.

Stocks of P2 and P18 strains of PICV were prepared from spleen extracts after inbred strain 13 guinea pigs were sequentially infected with the PICV Munchique strain CoAn4763 either twice to generate the P2 strain or 18 times to generate the P18 strain of the virus [22], provided to us by J. Aronson (University of Texas Medical Branch). It has been shown that infection with the P2 PICV leads to a brief febrile reaction in guinea pigs that soon fully recover, while P18 infection causes a severe disease that mimics human LF infection [20,22]. We previously developed reverse genetics systems for both the avirulent P2 and virulent P18 parental strains of PICV [24,25] and demonstrated that recombinant PICV strains rP2 and rP18 reproduced the differential disease phenotypes in outbred Hartley guinea pigs as those seen with the parental P2 and P18 strains [24]. Using reverse genetics systems, we generated reassortant recombinant viruses (rS2L18 and rS18L2) by swapping the large (L) and small (S) segments between P2 and P18 strains. While only the rS18L2 strain that carried the S segment from P2 and the L segment from P18 showed reduced virus titers in vitro, both viral reassortants (rS2L18 and rS18L2) showed attenuated phenotypes in vivo [26], indicating that genetic factors on both viral genomic segments are essential to cause virulent infection in vivo. While disease phenotypes and viral titers of outbred Hartley guinea pigs infected with the recombinant PICVs have been reported previously [24,26], the impact of viral infection on organs and tissues has not been comprehensively characterized. In the current study, we report the daily body weight and rectal temperature of all infected guinea pigs in Figure 1, which shows that the majority of both the parental P18- and recombinant rP18-infected guinea pigs reached terminal points for euthanization at day 14 post infection (pi) and day 18 pi, respectively, whereas a small number of animals within these groups were alive and seemed healthy at the end point of the experiments (i.e., 18 dpi) when these animals were humanely euthanized. Table 1 lists the animal identification numbers (ID #s) from which tissues samples were collected for histopathological and IHC analyses. Additionally, the viremia levels at different time points throughout the infection were quantified for each of the infected animals and shown in Table 2.

### 2.2. Histopathological and Immunohistochemical Analyses of Tissue Sections Prepared from Representative Healthy Guinea Pigs after Injection with Either PBS or the Parental P2 PICV

**Figure 2:**
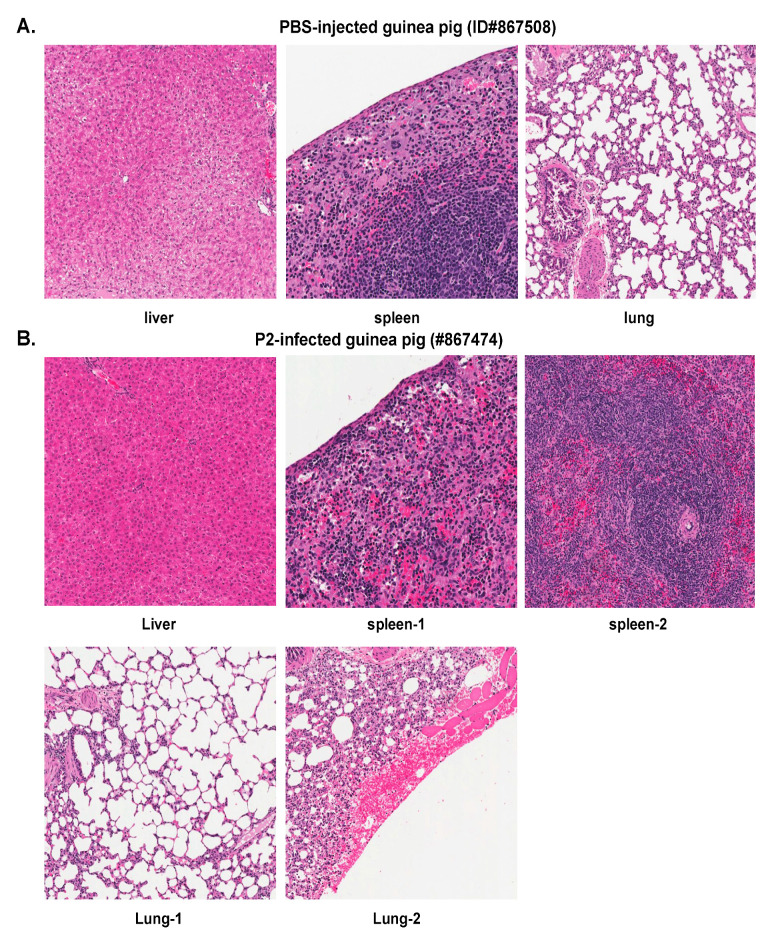
Histopathological findings of non-virulent PBS mock- and P2-infected outbred Hartley guinea pigs. H&E staining of tissue sections collected from organs of a representative PBS-injected animal (ID#867508) (**A**) and from a P2-infected animal (ID#867474) at 18 dpi (**B**).

A representative PBS-injected control animal (ID#867508) was found to be healthy during the entire duration of the experiment. Its body weight reached 139% at day 18 pi when compared to day 0 (Table 1). The H&E staining images of tissue sections from the liver, spleen, and lung of this animal are shown in Figure 2A, which show no significant histopathologic changes. Similar observations were made in the heart, kidney, gastrointestinal tract (GI), pancreas, adrenal gland, lymph node (LN), brain, and eye of this animal (data not shown). No IHC staining of PICV nucleoprotein (NP) antigen, the most abundantly expressed viral protein in infected cells and tissues, was detected in any of these tissue samples (data not shown), which was similar to the IHC images of tissues collected from the P2-infected animals as described below.

**Figure 3:**
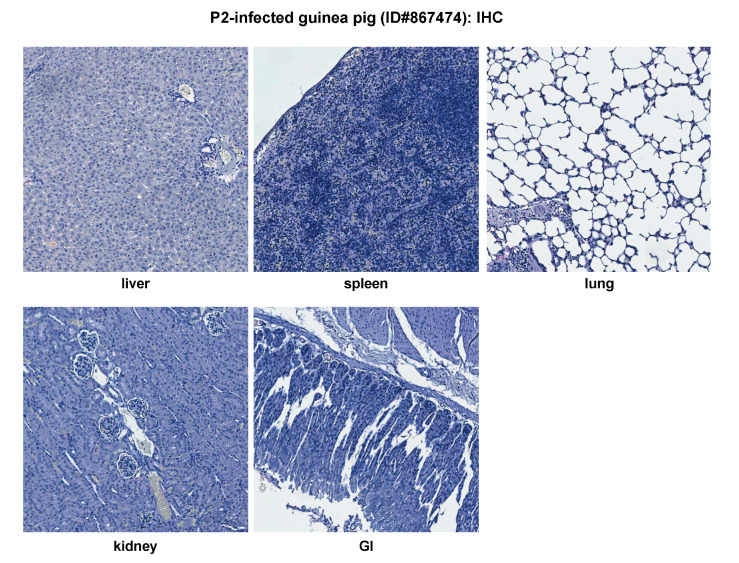
Immunohistochemical (IHC) staining for viral nucleoprotein (NP) antigens of a representative avirulent P2-infected outbred Hartley guinea pig (ID#867474) at 18 dpi. GI, gastrointestinal tract.

P2 generally induces a brief febrile reaction (average 1 day of fever) in outbred Hartley guinea pigs [24]. We analyzed a representative P2-infected outbred guinea pig (ID#867474) that experienced fever from day 6 to 9 pi with a rectal temperature > 39.5 °C. This is the maximal duration of fever observed for P2 infections. The animal showed slight body weight loss from day 8 to 10 pi, which grew to 132% on day 18 (a set end point of the experiment) as compared to day 0. The viremia level was found to be below the limit of detection (<200 PFU/mL) throughout the experiment (Table 2). The H&E images of liver, spleen, and lung are shown in Figure 2B. Although there are foci of hemorrhage on the pleural surface, no significant histopathologic changes are seen in the lung (Figure 2B). Similarly, no significant histopathologic changes were observed in other organs analyzed, including heart, kidney, GI, pancreas, adrenal gland, LN, brain, and eye (data not shown). No NP viral antigens were detected by IHC in any of the tissue samples examined, including liver, spleen, lung, kidney, and GI (Figure 3). These data demonstrate that the non-virulent P2 strain caused a brief and self-resolved disease in outbred Hartley guinea pigs and was completely cleared from the body of the infected animals.

### 2.3. Histopathological and Immunohistochemical Analyses of Tissue Sections Prepared from a Representative Moribund Guinea Pig Due to the Parental P18 PICV Infection

Outbred Hartley guinea pigs infected with the virulent P18 strain of PICV exhibited severe HF-like disease reminiscent of human LF and other AHFs [20,22]. A representative P18-infected guinea pig (ID#867480) started to develop a febrile reaction after 5 dpi, and its body weight loss became evident starting at day 6 pi. It was euthanized at day 14 pi after the animal reached a set of parameters, such as hypothermia and lost 26% of body weight (Table 1). The viremia level grew rapidly from 360 pfu/mL on day 6 pi to 4 × 10^6^ pfu/mL on day 14 pi (Table 2).

**Figure 4:**
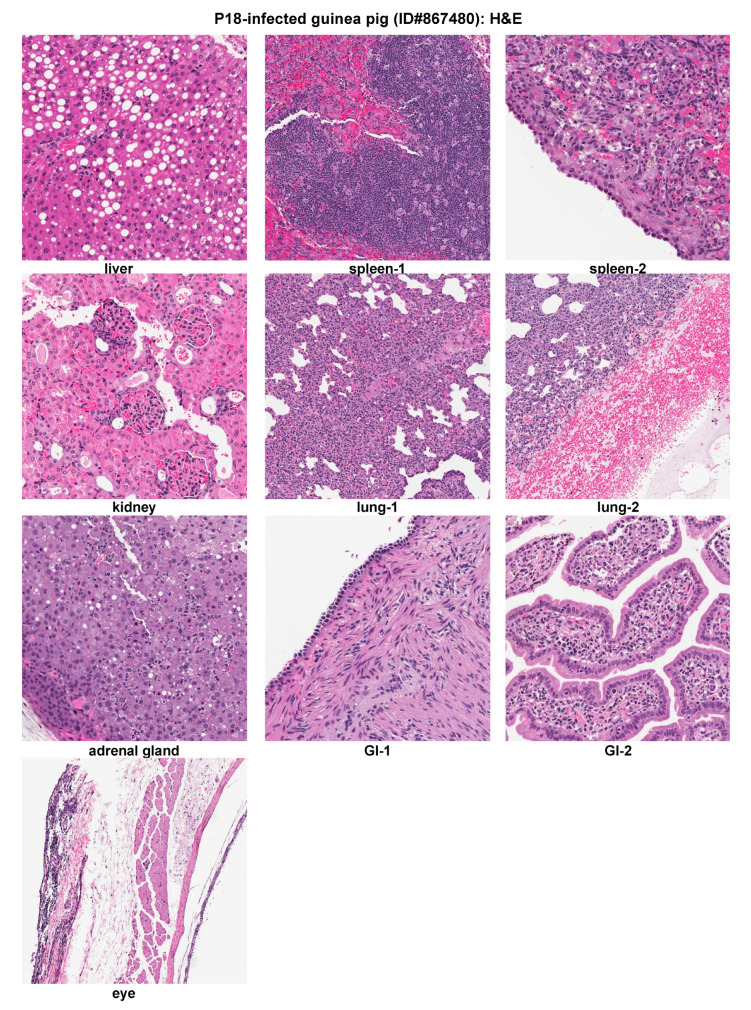
Histopathological findings of a representative virulent P18-infected outbred Hartley guinea pig. H&E staining of tissue sections collected from organs of a representative animal (ID#867480). GI, gastrointestinal tract.

Histopathologic examination was conducted on H&E staining of tissue sections of this representative animal (Figure 4). The liver showed severe fatty metamorphosis with foci of hepatocellular necrosis. The spleen showed sinusoidal congestion, focal lymphoid hyperplasia, and hyperplastic mesothelial cells on the serosal surface. The kidney showed mesangial cells proliferation in the glomeruli. The lung showed a moderate to intense increase of interstitial and peribronchiolar inflammatory infiltrates. Prominent pleural hemorrhage was also noticed. Hyperplastic mesothelial cells were present on the pleural surface. The adrenal gland showed focal necrosis and inflammatory infiltrates in the cortex. The GI showed focal submucosal edema, increased inflammatory infiltrates in lamina propria, and hyperplastic mesothelial cells on the serosal surface. The eye showed foci of inflammatory infiltrates in connective tissue. However, no significant histopathologic changes were seen in heart, pancreas, and skin (data not shown).

**Figure 5:**
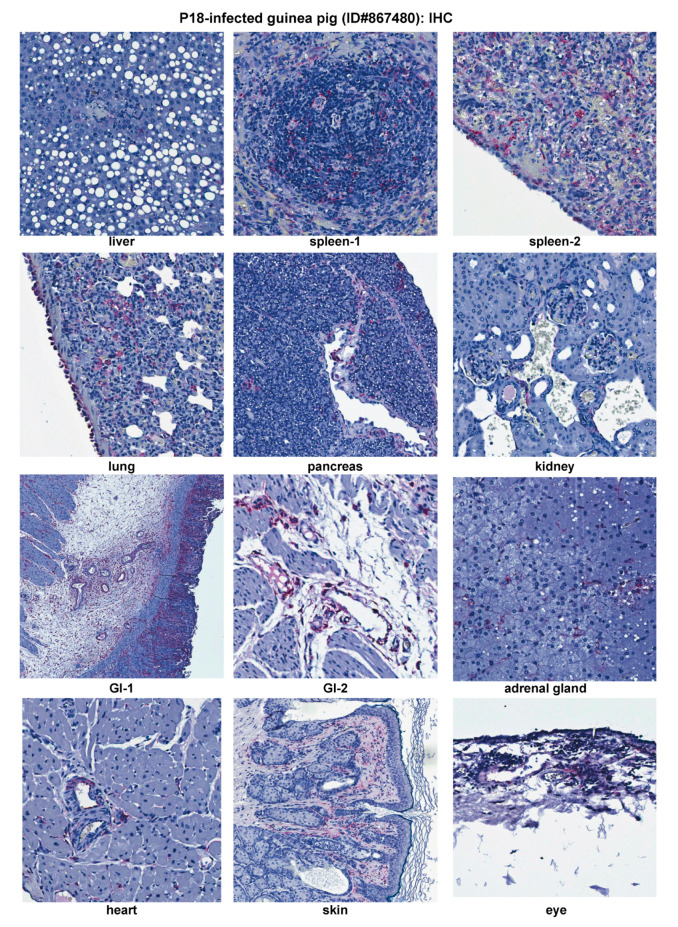
IHC staining for viral nucleoprotein (NP) antigens of a representative virulent P18-infected guinea pig (ID#867480). GI, gastrointestinal tract.

IHC demonstrated immunostaining of viral NP antigens in endothelial cells of all the tissues examined (Figure 5). In addition to endothelial cells, immunostaining in a variety of cells in different organs of the infected animal is also observed (Figure 5). These cells include (1) sinusoidal lining cells in liver and hepatocytes, especially those in periportal areas and necrotic foci; (2) dendritic cells in lymphoid follicles of spleen and mesothelial cells on serosa; (3) alveolar macrophages in lung and pleural mesothelial cells; (4) Islet cells of Langerhans and connective tissue in pancreas; (5) mesangial cells in kidney glomeruli; (6) adrenal cortical cells; (7) cells in the submucosa and lamina propria of gastrointestinal tract and mesothelial cells on the serosal surface; (8) interstitial myofibroblasts in heart and mesothelial cells on pericardium; (9) epithelial cells in dermis, dendritic cells and fibroblasts in dermis, and dermal appendages of the skin; and (10) fibroblasts in the connective tissue and inflammatory foci. In contrast, the brain was the only organ not showing any significant viral NP antigens, except for some rare immunostaining in endothelial cells of small vessels in the brain (data not shown). Taken together, these data demonstrate that virulent P18 virus involves in almost all tissues at relatively high levels and can infect many different cell types, such as macrophages, dendritic cells, endothelial cells, epithelial cells, mesothelial cells, fibroblasts, hepatocytes, and other specialized cells.

### 2.4. Histopathological and Immunohistochemical Analyses of Tissue Sections Prepared from a Representative Moribund Guinea Pig Due to the Recombinant rP18 PICV Infection

We next characterized a representative outbred Hartley guinea pig infected with the recombinant rP18 virus generated from reverse genetics technique and after plaque purification [24,26]. This representative animal (ID#867488) developed fever that started at day 5 pi and significant body weight loss started at day 9 pi, and was euthanized at day 18 pi when it showed signs of hypothermia and lost 20% of body weight (Table 1). The viremia level was determined at 1.8 × 10^6^ pfu/mL on day 18 pi, which is similar to that in the moribund animal infected with the parental P18 virus (Table 2).

**Figure 6:**
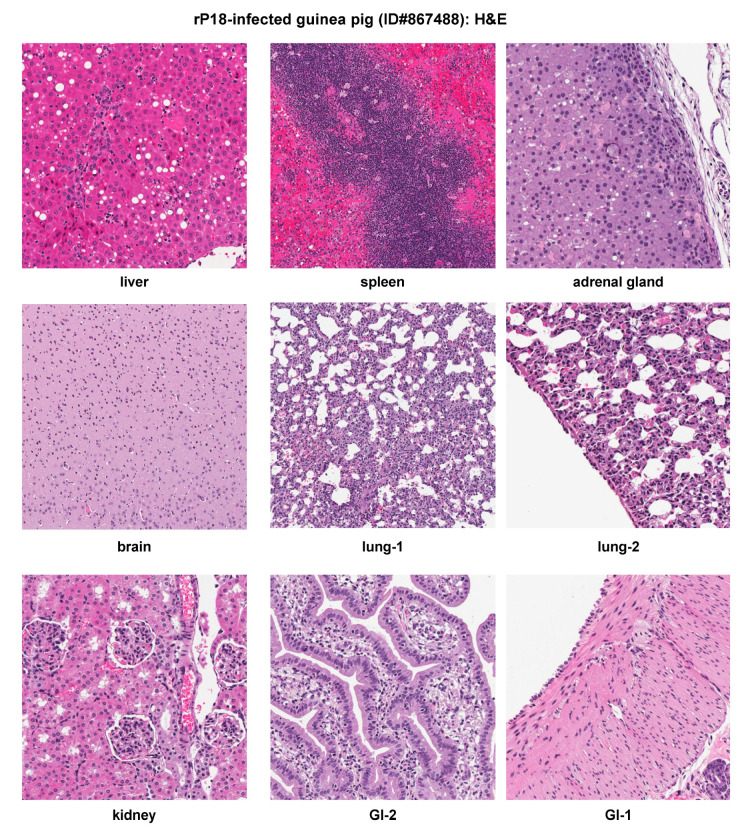
Histopathological findings of a representative virulent rP18-infected outbred Hartley guinea pig. H&E staining of tissue sections collected from organs of a representative animal (ID#867488). GI, gastrointestinal tract.

Significant histopathologic findings were observed in the liver, spleen, adrenal gland, lung, kidney, and GI, while the brain showed mild gliosis with no prominent inflammatory infiltrates or neuronal necrosis (Figure 6). These pathological changes in the rP18-infected guinea pig were very similar to the P18-infected one (Figure 4), with only some minor differences. For example, the rP18-infected animal showed less inflammatory infiltrates in the lungs and in the lamina propria, less severe fatty metamorphosis, but more necrosis and calcification in the liver (Figure 6). These minor differences are most likely due to some inherent variations of the individual outbred animal used in the experiment (Figure 1 and Table 1), but are not necessarily due to any intrinsic differences between the P18 and rP18 PICVs. In fact, we have shown that P18 and rP18 PICVs produce a similar degree of virulence in different groups of infected outbred Hartley guinea pigs [24], with the rP18 PICV causing a more consistent disease outcome in outbred guinea pigs [27].

**Figure 7:**
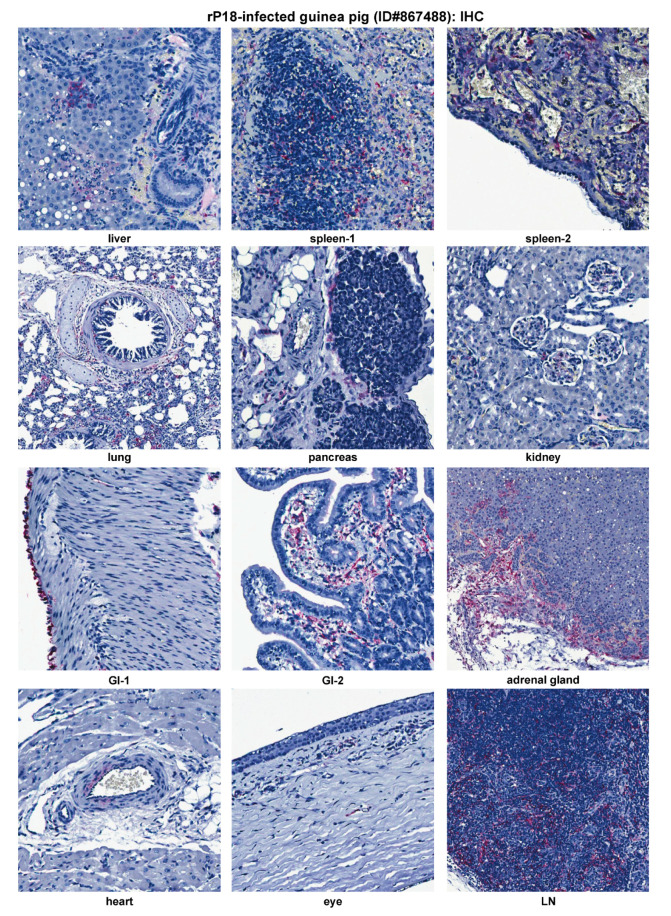
IHC staining for viral nucleoprotein (NP) antigens of a representative virulent rP18-infected guinea pig (ID#867488). GI, gastrointestinal tract. LN, lymph node.

Similar to P18 PICV infection (Figure 5), viral NP antigens were detected at relatively high levels in various extraneuronal tissues (liver, spleen, lung, pancreas, kidney, GI tract, adrenal gland, heart, LN, and eye) by IHC (Figure 7). Viral distribution in each of these tissues was similar between the P18 and rP18 PICV infections. IHC NP staining of LN was conducted for rP18-infected guinea pig only, in which viral antigens were detected in the follicular dendritic cells and endothelial cells in sinus (Figure 7).

Taken together, both P18 and rP18 PICVs caused virulent and multisystemic infections of outbred Hartley guinea pigs that showed significant pathological changes in various organs and tissues, similar to those seen in LF and other AHFs [28].

### 2.5. Histopathological and Immunohistochemical Analyses of Tissue Sections Prepared from a Representative Guinea Pig Infected with a Recombinant rS2L18 Reassortant Virus

**Figure 8:**
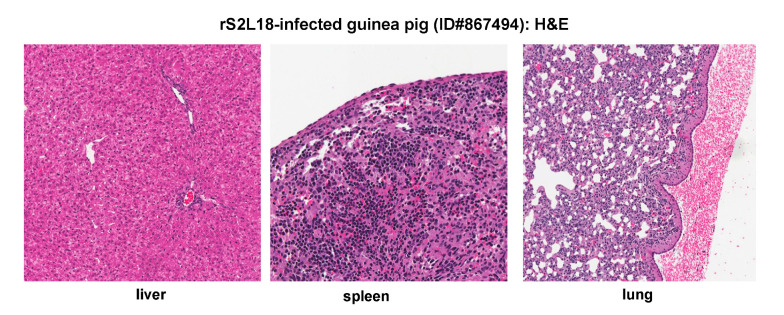
Histopathological findings of a representative reassorted rS2L18 virus-infected outbred Hartley guinea pig. H&E staining of tissue sections collected from organs of a representative animal (ID#867494).

We have previously shown that the rS2L18 PICV, which is a recombinant reassortant PICV with the P2 S segment and the P18 L segment, exhibits similar growth kinetics as the rP18 PICV in vitro but shows a much reduced degree of virulence in infected outbred Hartley guinea pigs [26]. A representative outbred guinea pig infected with the rS2L18 PICV (ID#867494) experienced febrile illness starting from day 6 pi to 10 pi (duration of fever = 5 days), showed a brief period of body weight loss from day 9 pi to 10 pi, but then recovered from the infection and was healthy at the set day of euthanization (day 18) with its body weight was at 116% of that at day 0 (Figure 1 and Table 1). The viremia levels on any given dates were below the limit of detection (<200 PFU/mL) (Table 2). No significant histopathologic changes were observed in the analyzed tissues including liver, spleen, and lung, except for some foci of hemorrhage observed on the pleural surface of the lung (Figure 8), which was also seen in the P2-infected animal (Figure 2B). As expected, no immunostaining of viral NP antigens was detected in any of the tissue samples examined (data not shown). Thus, outbred Hartley guinea pigs infected with the rS2L18 reassortant virus appeared to clear the viral infection and made a full recovery.

### 2.6. Histopathological and Immunohistochemical Analyses of Tissue Sections Prepared from a Representative Guinea Pig Infected with a Recombinant rS18L2 Reassortant Virus

Unlike the rS2L18 genome reassorted PICV described above, the rS18L2 reassortant virus that carries the P18 S segment and P2 L segment showed similar in vitro growth kinetics as those of the rP2, which was 0.5–1 log lower than rP18, suggesting that the viral factors encoded on the P18 L segment (e.g., the RNA-dependent RNA polymerase L gene) might be responsible for the higher rate of viral genome replication than those encoded on the P2 L segment [29]. However, like the rS2L18 PICV, this rS18L2 reassortant virus caused an attenuated phenotype in vivo [26]. A presentative rS18L2-infected guinea pig with the most severe disease in this group of animals was selected for histopathological analysis. This animal (ID#867500) developed fever from day 6 to 13 (duration of fever = 8 days), experienced body weight loss from days 9 pi to 16 pi, and was euthanized at day 18 pi when its body weight was 102% of that at day 0 (Figure 1 and Table 1). The viremia level throughout the 18-day infection period was below the detection limit of <200 PFU/mL (Table 2).

**Figure 9:**
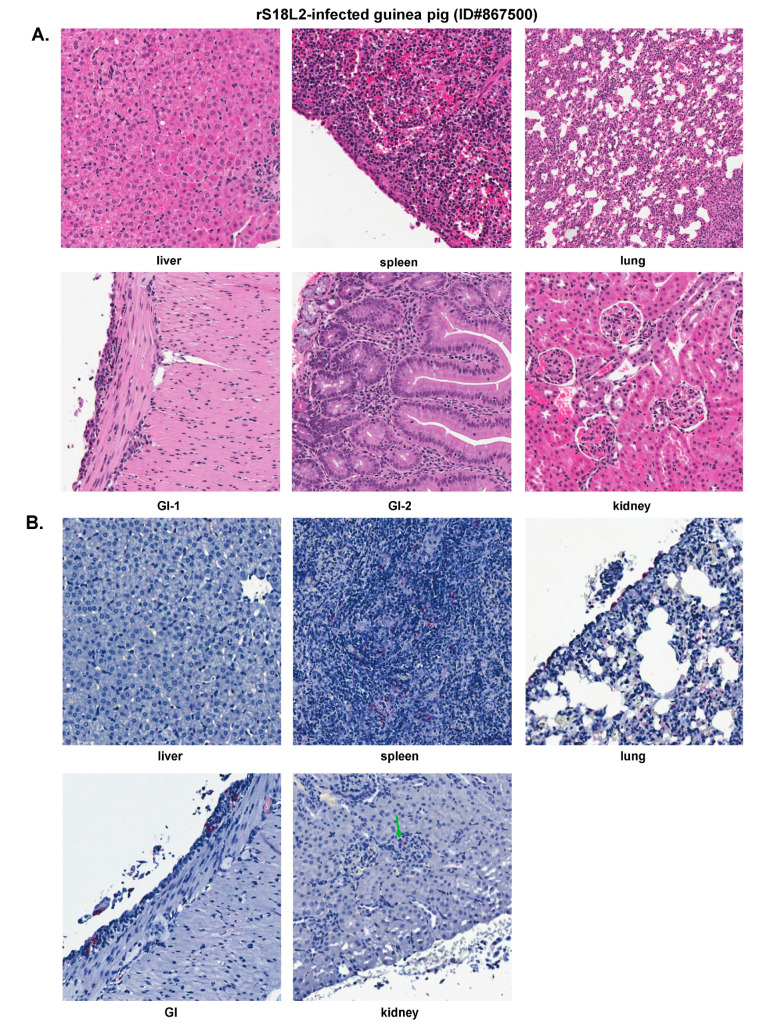
Histopathology (**A**) and IHC staining (**B**) of a representative reassorted rS18L12 virus-infected outbred Hartley guinea pig (ID#867500). A green arrow indicates positive IHC staining in the kidney. GI, gastrointestinal tract.

This representative rS18L2-infected guinea pig showed some histopathologic changes that were less severe than those observed in the P18- or rP18-infected outbred animals (Figure 9A). The liver showed scattered hepatocellular necrosis but not fatty metamorphosis. The spleen showed sinusoidal congestion and occasional hyperplastic mesothelial cells on the serosal surface. The lung showed a mild increase of interstitial and peribronchiolar inflammatory infiltrates. The GI tract showed small foci of increased inflammatory infiltrates in the lamina propria and focal hyperplastic mesothelial cells on the serosal surface. The kidney showed focal mild mesangial cells proliferation in the glomeruli.

Viral NP antigens were detected in endothelial cells, mesothelial cells, macrophages, and connective tissue cells in various organs (Figure 9B), but the amount of viral antigens were much less than those in P18- or rP18-infected groups of animals (Figure 5 and Figure 7). Positive IHC staining was detected in the mesothelial cells on the serosal surface of the liver, but was not detected in the parenchyma. In the spleen, there was scattered positive IHC staining in the endothelial cells, lymphoid follicle, and mesothelial cells. The lung showed rare positive immunostaining in interstitium and scattered staining in pleural mesothelial cells. Viral NP antigens were also detected in the GI tract, with positive staining seen in the focal mesothelial cells on the serosal surface and connective tissue but no prominent staining in the lamina propria or other locations. Rare positive staining was detected in the glomerular mesangial cells and endothelial cells in the kidney.

Collectively, the rS18L2-infected guinea pig showed a modest level of pathology in different tissues of the infected organs and an incomplete degree of virus clearance at the time of examination (18 dpi) (Figure 9). These findings are consistent with a less severe disease caused by the rS18L2 reassorted viral infection in outbred Hartley guinea pigs than those observed in outbred animals infected the highly virulent P18 or rP18 PICV (Table 1 and Table 2) [26]. The rS18L2-infected animal was able to control systemic infection, but took a longer period of time to clear the viruses than those outbred animals infected by either the P2 or rSL2L18 viruses.

## 3. Discussion

Similar to the work described in this current study, previous work has demonstrated LF-like clinical diseases in inbred Strain 13 guinea pigs infected with a guinea pig-adapted strain of PICV [20,21,22]. It takes a minimum of four sequential passages of PICV in the inbred animals to result in 100% uniform lethality with as low as 3 PFU of the virus used in the infection [22,23]. Infected animals are hypoactive and lethargic, with ruffled fur, decreased food/water intake, rapid and shallow breathing, slobbering, and significant weight loss before becoming moribund [21,23]. Viremia is detected by 2 dpi and steadily increases until death typically at 16 dpi with serum viral titers over 5 log_10_ PFU/mL [21,22,23]. Infected guinea pigs show marked levels of leukopenia at ~13 dpi and transient neutrophilia [22]. Further serologic analysis indicates impaired platelet function as evidenced by a decreased level of coagulation factors and thrombocytopenia [23]. White blood cell and platelet levels are also low, while hematocrit increases [23]. Liver AST enzyme levels are found to be elevated starting at ~9 dpi [22]. Gross and histopathological analyses show that the livers are enlarged and yellow-brown in color with moderate to severe hepatocellular necrosis with significant fatty changes but with only few inflammatory cells [20,21,22]. The red pulp of the spleen is engorged with fibrinous deposits, mild necrosis, and erythrophagocytic macrophages [20,21,22]. Pancreas samples contain acinar tissue lesions that are atrophied and vacuolated and replaced by fatty tissue, whereas the islets remain undamaged [21,22]. Lung samples show moderate to severe interstitial multifocal pneumonia with thickened alveolar septae and an influx of macrophages as well as perivascular edema and mild, nonspecific lesions [20,21,22]. Necrotic epithelial cells of the respiratory and gastrointestinal tract mucosa are noticeable [20,23], with lymphoid tissue featuring scattered necrotic regions and in the bone marrow but with a decrease number of mature granulocytes [23]. Similarly, the gastrointestinal tract shows foci of necrosis with histiocytic infiltration and the thymus is so degraded the cortex is almost absent [20,21]. Viral antigen is also detected in the salivary and adrenal glands as well as in the kidney with the infected adrenal glands showing small regions of tissue degeneration [21,22]. Similar to LASV infection, brain samples of PICV-infected inbred guinea pigs did not show significant histopathologic changes, although viral NP antigen can be detected in several small vessels, likely from the viremia [21,22], which are similar to our observation of outbred animal infected with a virulent strain of PICV (P18 or rP18). As this inbred model infected with the guinea pig-adapted strain of PICV is remarkably similar to the inbred animals infected with LASV, this PICV-inbred guinea pig model has served as an excellent non-high containment model of LF [22]. It is important to note, however, that while inbred guinea pigs may be a useful model for LF, they are not readily available and lack genetic diversity mimicking human populations. On the contrary, outbred Hartley guinea pigs are commercially available and have a diverse genetic background.

Our detailed analysis of histopathology and viral immunostaining of a wide range of tissues prepared from organs of outbred Hartley guinea pigs infected with a guinea pig-adapted strain of PICV (P18) or a recombinant virus (rP18) generated by reverse genetic technique shows that these virulent PICV strains can accurately replicate the histopathological findings in various infected extraneuronal tissues that correlate well with humans or other animals infected with a pathogenic strain of LASV. The similarity of pathology and viral antigen distribution in tissues/organs of virulent PICV infection of the outbred Hartley guinea pig described in the current study and those of lethal human LF supports the use of this Hartley outbred guinea pig as a relevant surrogate model to model LF disease and to test vaccines and antivirals against it. It is important to point out that LASV infections of inbred Strain 13 guinea pigs have previously been described [14,15,30] that shows similar disease phenotype, yet with some unique differences as described below.

Infection of the inbred Strain 13 guinea pigs with the known human virulent LASV Josiah strain has demonstrated at least 90% lethality [14,15,30]. The Z-132 strain of LASV is 100% lethal in these animals, but the Soromba-R strain of LASV show variable degrees of virulence (0–57% lethality) with a very mild clinical disease [31,32]. Signs of clinical disease in virulent LASV infections include weight loss, fever, ruffled fur, hunched posture, altered mentation, and conjunctivitis [13,14,15]. Infected animals become moribund typically between 10 dpi and 15 dpi [14]. A persistent viremia develops high titers rapidly (around 56 dpi) and peaks around 10 dpi with highest viral load being identified in the lung, followed by the spleen, pancreas, lymph nodes, adrenal glands, kidneys, salivary glands, liver, and heart [13,30,32]. Unlike our outbred Hartley guinea pigs infected with the virulent P18 or rP18 virus, virulent LASV is detectable in brain samples, but at such low titers that it is assumed to be from the blood infection [30]. Histologic findings include the presence of viral NP antigens, which are typically associated with hepatitis, hepatic cell death, hair follicle epithelium cell death, and adrenal cortical necrosis [13]. Laryngitis and heterophilic tracheitis have been noted in guinea pigs infected via the aerosol route [13]. Similar to our outbred animal model of virulent P18 or rP18 PICV infection, interstitial pneumonia was found in all moribund inbred animals infected with virulent strains of LASV with an increase of mononuclear cells, especially macrophages, surrounding the blood vessels and airways [13,14,30,32]. Edema and hemorrhage was also noted in the lungs of virulent LASV infections, with an expansion of the alveolar septae and a mild to moderate inflammation in portal and sinusoidal regions of the liver that show evidence of vacuolar degeneration and necrosis [13,14,32]. Half of the inbred guinea pigs infected with virulent LASV develop acute necrotizing nephritis and mild myocarditis or mild to moderate endocarditis and pancarditis [13,14,30], with heart-related phenomena not seen in human clinical cases [5]. Five of eight inbred guinea pigs infected with virulent LASV develop minimal hepatitis and necrotizing lesions of the spleen [30] that show mild heterophilic splenitis [13,32]. The red pulp of the spleen shows perifollicular to diffuse mononuclear cell proliferation [13]. Lymphocytolysis and lymphoid depletion has been noted in the white pulp of the spleen, thymus, and lymph nodes in virulent LASV infections [13,14]. The pancreas shows acinar cell atrophy as well as other architectural changes [13]. Renal tubular degeneration and peritonitis are also infrequently noted in these animals [14]. Viral NP antigen has been detected in the reproductive tissues of both male and female inbred guinea pigs infected with the virulent strains of LASV, with males also showing evidence of mild epididymitis [13]. Serum analysis of these animals detect an elevated level of ALT and heterophils with a decrease levels of platelets and blood lymphocytes [13]. Additionally, while exhibiting evidence of viral infection, the adrenal glands of virulent LASV-infected inbred animals show minimal damage [13,30]. Similar to our outbred guinea pigs infected with the virulent P18 or rP18 PICVs, virulent LASV-infected inbred animals develop noticeable petechial rashes [13].

A unique clinical feature of Strain 13 guinea pigs infected with virulent LASV is the presentation of conjunctivitis due partly to infection of the eye [15], which is consistent with clinical human LASV infections that have been known to exhibit conjunctival edema and conjunctivitis in acute cases of the infection and can lead to transient blindness in survivors. It is noteworthy that LASV has been detected in the aqueous humor of the eye of infected rhesus macaques [15,33]. Viral antigen can be found in the endothelial cells of the sclera, deep to Descemet’s membrane; conjunctiva; and cornea of the eye [15]. Affected mesenchymal cells have been found in the ciliary body, iris, and filtration angle, as well as in neural crest cells and the perivascular connective tissue [15]. Interestingly, a large proportion (8/9) of lethal LASV infections of inbred guinea pigs show mild peripheral corneal neovascularization with viral antigen present in five of those animals, and six of nine of them also feature evidence of mild conjunctival hemorrhage [15].

Unless the Josiah strain of LASV that has been adapted in guinea pigs [13] is used (as described in some detail below), infection of outbred Hartley guinea pigs with a non-guinea pig-adapted LASV Josiah strain results in only 30–67% lethality regardless of dosage of the virus being used in these experiments [5,30]. Regardless, all moribund animals show evidence of interstitial pneumonia [30], while viremia develops more slowly and to lower levels than the infected inbred Strain 13 animals, which allows the animals to clear the infection by 21 dpi [5,30]. High viral titers have been noted in the lung, kidney, liver, heart, bladder, thymus, adrenal gland, spleen, lymph nodes, uterus, and ovary, and some low levels of the virus are also detectable in the urine, blood, and ascetic and pleural fluid [5]. Histologic analysis indicates lung samples of LASV-infected outbred Hartley guinea pigs with pulmonary edema and microscopic lesions [5]. Similar to the infected inbred animals, the heart tissues of the infected outbred animals show evidence of mild to moderate myocarditis [5]. Hepatocytes and myocardial fibers also show some evidence of calcification [5].

A Josiah strain of LASV adapted in guinea pigs through serial passages can lead to a uniform lethality in the Hartley outbred guinea pig with only 1 in 18 infected guinea pigs surviving the infection [34,35]. These infected animals develop febrile illness around 6–9 dpi, followed by respiratory distress and hypothermia [35,36]. Similar to our outbred animals infected with the virulent P18 or rP18 PICV, outbred animals infected with the guinea pig-adapted strain of LASV show rapid weight loss, ranging from 8% to over 20%, which is a typical humane euthanasia criterion, and died at an average of 15 dpi [34,35,36]. These animals also show an unstable gait, a noticeable lack of grooming with associated rough/ruffled fur, delayed responsiveness/lethargy, recumbency, and a reddening of the footpads and ears [34,35]. Viral titers have been detected in the serum, spleen, liver, and lung of these animals [35,36]. Histologic analysis of the liver indicates lymphohistiocytic hepatitis and hepatocellular degeneration, whereas spleen samples shows evidence of sinus histiocytosis and lung samples feature interstitial pneumonia [35].

While different animal species have been developed as animal models of LF [4], they all have certain constraints. For example, the use of NHPs, such as marmosets or rhesus macaques, can incur exorbitant expenses of high containment BSL4 facilities and experienced lab personnel (veterinarians) needed to conduct LASV studies. Laboratory mice, on the other hand, are relatively inexpensive, yet they are generally resistant to LASV infection. Therefore, other small and less expensive animals, such as inbred/outbred guinea pigs infected with the guinea pig-adapted strain of PICV, have been explored as potential alternative models of LF. As demonstrated in the current study, outbred Hartley guinea pigs infected with a virulent strain of PICV can produce a disease that is remarkably similar to inbred animals infected with LASV or in human LF, which makes this PICV-guinea pig model an attractive model for understanding LF disease pathogenesis and for testing candidate vaccines and therapeutics.

## 4. Materials and Methods

### 4.1. In Vivo Experiment

Experimental infection of outbred Harley guinea pigs, purchased from Charles River Laboratories, were carried out as previous described [24,26] and followed the approved protocols from the Institutional Animal Care and Use Committee (IACUC) at Emory University. Healthy outbred Hartley guinea pigs (300–350 g) (N = 6) were infected intraperitoneally (IP) with 10,000 PFU of the respective PICV strains (P2, rP2, P18, or rP18). Rectal temperature and body weight were measured daily and up until day 18 post infection (18 dpi), which is the last day of the experiments. Fever is defined as rectal temperature of >39.5 °C. Blood was collected at certain time points, and viremia was measured by plaque assay as described below. Guinea pigs were declared moribund and euthanized if body weight decreased by 25% and rectal temperature fell below 38.5 °C. At terminal points or at 18 dpi, guinea pigs were euthanized, and organs were removed and preserved in formalin before tissue sectioning.

### 4.2. Histopathological and Immunohistochemical Analyses

Tissue samples collected from PICV-infected guinea pigs were fixed in 4% formaldehyde, embedded in paraffin, sectioned, stained with hematoxylin and eosin (H&E), and examined microscopically for histopathological changes. For immunohistochemical staining of PICV antigens, paraffin-embedded tissue sections were subjected to antigen retrieval followed by staining with mouse anti-PICV serum. Sequential treatment with biotinylated secondary antibodies, alkaline phosphatase-conjugated streptavidin, and napthol fast red was conducted according to the manufacturer’s protocol (LSAB2 Universal Alkaline Phosphatase kit, DAKO, Carpinteria, CA, USA), and followed by counterstaining in Meyer’s hematoxylin [37].

### 4.3. PICV Plaque Assay

Vero cells were seeded into six-well plates at 90% to 100% confluency and infected with 0.5 mL of 10-fold serial dilutions of the respective PICV in MEM complete media for 1 h at 37 °C. After removing the medium, the cells were incubated in fresh media supplemented with 0.5% agar and cultured for 4 days at 37 °C. Plaques were stained overnight with diluted neutral red solution (1:50) in 0.5% agar–MEM–10% FBS.

## Figures and Tables

**Table 1:** Guinea pigs infected with different virulent (P18 or rP18) and avirulent (P2 or rP2) PICV strains or PBS control and their disease phenotypes.

Guinea Pig ID	Viruses	Fever, BW Loss ^1^	DoE ^2^	Health, BW at DoE ^3^
867508	PBS	No fever, no BW loss	18 dpi	Healthy, BW 139%
867474	P2	Fever d6 to d9, BW loss d9 to d10	18 dpi	Healthy, BW 132%
867480	P18	Fever d5 to d13, BW loss since d8	14 dpi	Moribund, BW 74%
867488	rP18	Fever d5 to d17 BW loss since d9	18 dpi	Moribund BW 80%
867494	rS2L18	Fever d6 to d10 BW loss d9 to d10	18 dpi	Healthy, BW 116%
867500	rS18L2	Fever d6 to d13 BW loss d9 to d16	18 dpi	Healthy, BW 102%

^1^ daily observation of rectal temperature and body weight (BW). ^2^ DoE, date of euthanization, either on 18 dpi or when reaching terminal points. ^3^ BW at the date of euthanization was normalized to its BW at day 0 (set as 100%). TP, terminal points.

**Table 2:** Viremia levels in outbred Hartley guinea pigs infected with virulent (P18 or rP18) or avirulent (P2 or rP2) strains of PICV following different days of the infection.

Guinea Pig ID	Viruses	6 dpi (PFU/mL)	12 dpi (PFU/mL)	TP ^1^ or 18 dpi (PFU/mL)
867474	P2	BD	BD	BD
867480	P18	360	3.4 × 10^4^	4 × 10^6^
867488	rP18	NA	NA	1.8 × 10^6^
867494	rS2L18	BD	BD	BD
867500	rS18L2	BD	BD	BD

^1^ TP, terminal points. BD, below the limit of detection plaque assay (<200 pfu/mL). NA, not analyzed.

## References

[1] (1972). Lassa fever. Br. Med. J..

[2] CDC (2019). Lassa Fever. https://www.cdc.gov/vhf/lassa/index.html.

[3] Ibekwe T.S., Okokhere P.O., Asogun D., Blackie F.F., Nwegbu M.M., Wahab K., Omilabu S.A., Akpede G.O. (2010). Early-onset sensorineural hearing loss in Lassa fever. Eur. Arch. Oto-Rhino-Laryngology.

[4] Sattler R.A., Paessler S., Ly H., Huang C. (2020). Animal models of lassa fever. Pathogens.

[5] Walker D.H., Wulff H., Lange J.V., Murphy F.A. (1975). Comparative pathology of Lassa virus infection in monkeys, guinea-pigs, and Mastomys natalensis*. Bull. World Heal. Organ..

[6] Oestereich L., Lüdtke A., Ruibal P., Pallasch E., Kerber R., Rieger T., Wurr S., Bockholt S., Pérez-Girón J.V., Krasemann S. (2016). Chimeric mice with competent hematopoietic immunity reproduce key features of severe lassa fever. PLoS Pathog..

[7] Rieger T., Merkler D., Günther S. (2013). Infection of type I interferon receptor-deficient mice with Various Old World Arenaviruses: A model for studying virulence and host species barriers. PLoS ONE.

[8] Yun N.E., Poussard A.L., Seregin A.V., Walker A.G., Smith J.K., Aronson J.F., Soong L., Paessler S., Smith J.K., Smith J.N. (2012). Functional interferon system is required for clearance of Lassa Virus. J. Virol..

[9] Yun N.E., Seregin A.V., Walker D.H., Popov V.L., Walker A.G., Smith J.N., Miller M., De La Torre J.C., Borisevich V., Fair J.N. (2013). Mice lacking functional STAT1 are highly susceptible to lethal infection with Lassa Virus. J. Virol..

[10] Yun N.E., Ronca S., Tamura A., Koma T., Seregin A.V., Dineley K.T., Miller M., Cook R., Shimizu N., Walker A.G. (2015). Animal model of Sensorineural hearing loss associated with Lassa Virus infection. J. Virol..

[11] Oestereich L., Rieger T., Lüdtke A., Ruibal P., Wurr S., Pallasch E., Bockholt S., Krasemann S., Muñoz-Fontela C., Günther S. (2015). Efficacy of Favipiravir alone and in combination with ribavirin in a lethal, immunocompetent mouse model of Lassa Fever. J. Infect. Dis..

[12] Flatz L., Rieger T., Merkler D., Bergthaler A., Regen T., Schedensack M., Bestmann L., Verschoor A., Kreutzfeldt M., Bruck W. (2010). T Cell-dependence of lassa fever pathogenesis. PLoS Pathog..

[13] Bell T.M., Shaia C., Bearss J.J., Mattix M.E., Koistinen K.A., Honnold S.P., Zeng X., Blancett C.D., Donnelly G.C., Shamblin J.D. (2016). Temporal progression of lesions in guinea pigs infected with lassa virus. Veter Pathol..

[14] Cashman K.A., Smith M.A., Twenhafel N.A., Larson R.A., Jones K.F., Allen R.D., Dai D., Chinsangaram J., Bolken T.C., Hruby D.E. (2011). Evaluation of Lassa antiviral compound ST-193 in a guinea pig model. Antivir. Res..

[15] Gary J.M., Welch S.R., Ritter J.M., Coleman-McCray J., Huynh T., Kainulainen M.H., Bollweg B.C., Parihar V., Nichol S.T., Zaki S.R. (2019). Lassa Virus Targeting of Anterior Uvea and Endothelium of Cornea and Conjunctiva in Eye of Guinea Pig Model. Emerg. Infect. Dis..

[16] Maruyama J., Manning J.T., Mateer E.J., Sattler R., Bukreyeva N., Huang C., Paessler S. (2019). Lethal infection of lassa virus isolated from a human clinical sample in outbred guinea pigs without adaptation. mSphere.

[17] Zapata J.C., Pauza C.D., Djavani M.M., Rodas J.D., Moshkoff D., Bryant J., Ateh E., Garcia C., Lukashevich I.S., Salvato M.S. (2011). Lymphocytic choriomeningitis virus (LCMV) infection of macaques: A model for Lassa fever. Antivir. Res..

[18] Remy M.M., Şahin M., Flatz L., Regen T., Xu L., Kreutzfeldt M., Fallet B., Doras C., Rieger T., Bestmann L. (2017). Interferon-γ-Driven iNOS: A molecular pathway to terminal shock in Arenavirus Hemorrhagic Fever. Cell Host Microbe.

[19] Trapido H., Sanmartín C. (1971). Pichindé virus, a new virus of the Tacaribe group from Colombia. Am. J. Trop. Med. Hyg..

[20] Aronson J.F., Herzog N.K., Jerrells T.R. (1994). Pathological and virological features of arenavirus disease in guinea pigs. Comparison of two Pichinde virus strains. Am. J. Pathol..

[21] Connolly B.M., Geyer S., Peters C.J., McPherson R.A., Barth J.F., Jenson A.B. (1993). Pathogenesis of pichinde virus infection in strain 13 guinea Pigs: An immunocytochemical, virologic, and clinical chemistry study. Am. J. Trop. Med. Hyg..

[22] Jahrling P.B., Hesse R.A., Rhoderick J.B., Elwell M.A., Moe J.B. (1981). Pathogenesis of a pichinde virus strain adapted to produce lethal infections in guinea pigs. Infect. Immun..

[23] Cosgriff T.M., Jahrling P.B., Chen J.P., Hodgson L.A., Lewis R.M., Green D.E., Smith J.I. (1987). Studies of the coagulation system in arenaviral hemorrhagic fever: Experimental infection of strain 13 Guinea Pigs with Pichinde Virus. Am. J. Trop. Med. Hyg..

[24] Lan S., Schelde L.M., Wang J., Kumar N., Ly H., Liang Y. (2009). Development of infectious clones for virulent and avirulent pichinde viruses: A model virus to study arenavirus-induced hemorrhagic fevers. J. Virol..

[25] Dhanwani R., Huang Q., Lan S., Zhou Y., Shao J., Liang Y., Ly H. (2018). Establishment of bisegmented and trisegmented reverse genetics systems to generate recombinant Pichindé viruses. Breast Cancer.

[26] Liang Y., Lan S., Ly H. (2009). Molecular determinants of pichinde virus infection of guinea pigs-a small animal model system for arenaviral hemorrhagic fevers. Ann. N. Y. Acad. Sci..

[27] Lan S., Shieh W.J., Huang Q., Zaki S.R., Ly H., Liang Y. (2020). Recombinant Pichinde virus infection of outbred Hartley guinea pigs as an improved surrogate small animal model for Lassa fever. Virulence.

[28] Walker D.H., McCormick J.B., Johnson K.M., Webb P.A., Komba-Kono G., Elliott L.H., Gardner J.J. (1982). Pathologic and virologic study of fatal Lassa fever in man. Am. J. Pathol..

[29] McLay L., Lan S., Ansari A., Liang Y., Ly H. (2013). Identification of Virulence Determinants within the L Genomic Segment of the Pichinde Arenavirus. J. Virol..

[30] Jahrling P.B., Smith S.A., Hesse R., Rhoderick J.B. (1982). Pathogenesis of Lassa virus infection in guinea pigs. Infect. Immun..

[31] Safronetz D., Mire C., Rosenke K., Feldmann F., Haddock E., Geisbert T., Feldmann H. (2015). A Recombinant vesicular stomatitis virus-based Lassa fever vaccine protects guinea pigs and macaques against challenge with geographically and genetically distinct Lassa Viruses. PLoS Neglected Trop. Dis..

[32] Safronetz D., Strong J.E., Feldmann F., Haddock E., Sogoba N., Brining U., Geisbert T.W., Scott D.P., Feldmann H. (2013). A recently isolated Lassa virus from Mali demonstrates atypical clinical disease manifestations and decreased virulence in Cynomolgus macaques. J. Infect. Dis..

[33] Walker D.H., Johnson K.M., Lange J.V., Gardner J.J., Kiley M.P., McCormick J.B. (1982). Experimental infection of rhesus monkeys with Lassa virus and a closely related arenavirus, mozambique virus. J. Infect. Dis..

[34] Cross R.W., Mire C.E., Branco L.M., Geisbert J.B., Rowland M.M., Heinrich M.L., Goba A., Momoh M., Grant D.S., Fullah M. (2016). Treatment of Lassa virus infection in outbred guinea pigs with first-in-class human monoclonal antibodies. Antivir. Res..

[35] Safronetz D., Rosenke K., Westover J.B., Martellaro C., Okumura A., Furuta Y., Geisbert J., Saturday G., Komeno T., Geisbert T.W. (2015). The broad-spectrum antiviral favipiravir protects guinea pigs from lethal Lassa virus infection post-disease onset. Sci. Rep..

[36] Stein D.R., Warner B.M., Soule G., Tierney K., Frost K.L., Booth S., Safronetz D. (2019). A recombinant vesicular stomatitis-based Lassa fever vaccine elicits rapid and long-term protection from lethal Lassa virus infection in guinea pigs. npj Vaccines.

[37] Butler J.C., Zaki S.R., Khabbaz R.F., Peters C.J. (1995). Hantavirus pulmonary syndrome. Infect. Dis. Clin. Pr..

